# Supplementary material for: Patterns of change in α and β taxonomic and phylogenetic diversity in the secondary succession of semi-natural grasslands in the Northern Apennines
Source: PeerJ. 2020 Mar 11;8:e8683. doi: 10.7717/peerj.8683 (PMC7071822; doi:10.7717/peerj.8683)
Supplement: Supplemental Information 1 — Appendix S1: Detailed methods for the molecular analyses and phylogenetic tree reconstruction. Appendix S2: Description of SDR indices calculation. Table S1: List of taxa used for the implementation of the multi-alignment. Table S2: List of species sampled and relative accession numbers in the GenBank. Table S3: Results of indicator species analysis (ISA). Figure S1: Phylogenetic tree of the plant species recorded in the 60 plots (figure). [file peerj-08-8683-s001.docx]

**Supplemental Materials for:**

**Patterns of change in α and β taxonomic and phylogenetic diversity in the secondary succession of semi-natural grasslands in the Northern Apennines**

Lorenzo Lazzaro^1^, Lorenzo Lastrucci^2^, Daniele Viciani^1^, Renato Benesperi^1^, Vincenzo Gonnelli^3^ & Andrea Coppi^1^

^1^Department of Biology, University of Florence, Via G. La Pira, 4, 50121 Florence, Italy;

^2^Natural History Museum, Botany, University of Florence, via G. La Pira, 4, 50121 Florence, Italy;

^3^Via Martiri della Libertà 1, 52036 Pieve S. Stefano, Italy.

Submitted to:

PeerJ — the Journal of Life and Environmental Sciences

Corresponding author: Lorenzo Lazzaro, Tel. +39 055 2757372, lorenzo.lazzaro@unifi.it

**Appendix S1** Detailed methods for the molecular analyses and phylogenetic tree reconstruction

Isolation of genomic DNA followed a modified 2×CTAB protocol (Doyle and Doyle, 1990). Amplification of the ITS region was performed using the primers ITS4 and ITS5 (White et al., 1990). Polymerase chain reactions were performed in a total volume of 25 μl containing 2.5 μl of 10× reaction buffer (Dynazyme II, Finnzyme, Espoo, Finland), 1.5 mM MgCl2, 10 pmol of each primer, 200 μM dNTPs, 1 U of TaqDNA polymerase (Dynazyme II) and 10 ng of template DNA. Reactions were performed in a MJ PTC-100 thermocycler (Peltier ThermalCycler, MJ Research, Waltham, Massachusetts, U.S.A.). Subsequently, 5 μl of each amplification mixture were analysed by agarose gel electrophoresis in TAE buffer (1.5% w/v) containing1 μg/ml ethidium bromide, by comparison with a known mass standard. After purification (Roche, Mannheim purification kit, Germany), the PCR reactions were quantified with a spectrophotometric method (Biophotometer, Eppendorf).

Automated DNA sequencing was performed from the PCR products using BigDye Terminator v.2 chemistry and an ABI310 sequencer (PE-Applied Biosystems, Norwalk, Connecticut, U.S.A.).

Original sequences were checked for homology with Blast (http://blast.ncbi.nlm.nih.gov/Blast.cgi) and edited with BioEdit v.7.0 (Hall, 1999). The few overlapping peaks at the same 1-bp position, possibly associated with heterozygous alleles or minor DNA polymerase errors, were translated following the IUPAC ambiguity code. All original sequences were deposited in GenBank and can be retrieved using the accession number in Appendix S2.

Due to missing amplification, five species (i.e. *Tamus communis*, *Pulmonaria picta*, *Helianthemum nummularium*, *Geranium rotondifolium* and *Colchicum lusitanicum*) were substituted by high-related proxy (*Dioscorea polystachya*, *Pulmonaria rubra*, *Helianthemum squamatum*, *Geranium pyrenaicum* and *Colchicum autumnale* respectively) in order not to lose important phylogenetic information.

As showed in Table S1, 10 taxa from Gnetales, Ginkgoales, Cycadales, Pinales and Cupressales were added to the multi-alignment in order to obtain a correct evolutionary position of *Juniperus communis* in the phylogeny reconstruction. Two taxa for Polypodiales and Salviniales were selected as outgroup.

**References**

Doyle, J.J. & Doyle, J.L. 1990. Isolation of plant DNA from fresh tissue. Focus 12: 13–15.

Hall, T.A. 1999. BioEdit: A user–friendly biological sequence alignment editor analysis program for windows 95/98/NT. Nucleic Acids Symposium Series 41: 95–98.

White, T.J., Bruns, T., Lee, S. & Taylor, J. 1990. Amplification and direct sequencing of fungal ribosomal RNA genes for phylogenetics. In: Innis, M.A., Gelfand, D.H., Sninsky, J.J. & White, J.W. (eds.) PCR protocols: A guide to methods and applications, pp. 315–322. New York: Academic Press.

**Appendix S2.** Description of SDR indices calculation

Calculations were computed using the computer program SDR Simplex (Podani, 2001). In particular, the three indices were calculated as follows (further details in Podani and Schmera, 2011).

Similarity (S) was calculated according to the Jaccard coefficient of similarity:

1) $S_{Jac}=a/n$

where *a* is the number of species shared by plots and *n* is total number of species.

Richness difference (*D*) was calculated as the ratio of the absolute difference between the species numbers of each site (*b, c*) and the total number of species, *n*:

2) $D= 1 - (a+ 2min\{b,c\})/n$

Finally, species replacement (*R*) was given by

3) $R= 2min\{b,c\}/n$

where in 2) e 3) *b* is the number of species present only in the first site and *c* is the number of species present only in the second site, so that *min*{*b,c*} is the smaller number between *b* and *c* representing the number of species in the less rich site that are replaced by new species in the richest.

**References**

Podani, J. & Schmera, D. 2011. A new conceptual and methodological framework for exploring and explaining pattern in presence–absence data. Oikos 120: 1625–1638.

**Table S1**. List of taxa used for the implementation of the multi-alignment.

| **Gymnosperms** | | |
| --- | --- | --- |
| **Specie** | **Family** | **Genbank accession number** |
| Chamaecyparis thyoides | Cupressaceae | AY283430 |
| Cryptomeria japonica | Cupressaceae | AF387522 |
| Taxus globosa | Taxaceae | MH267486 |
| Cephalotaxus sinensis | Taxaceae | EF660591 |
| Cycas parvula | Cycadaceae | KP117107 |
| Zamia lindenii | Zamiaceae | AF531245 |
| Ginkgo biloba | Ginkgoaceae | EF372233 |
| Gnetum leyboldii | Gnetaceae | AY449617 |
| Ephedra sinica | Ephedraceae | GU968566 |
| Pseudotsuga wilsoniana | Pinaceae | AF041351 |
| **Outgroup** | | |
| **Specie** | **Family** | **Genbank accession number** |
| Asplenium scolopendrium | Aspleniaceae | JX475163 |
| Asplenium aethiopicum | Aspleniaceae | JX475148 |
| Azolla caroliniana | Salviniaceae | JX297319 |
| Azolla filiculoides | Salviniaceae | JX297318 |

**Table S2.** List of species sampled in experimental plots. Nomenclature follows Conti et al., 2005. Genbank accession number allows to retrieve the species in the GenBank at <http://www.ncbi.nlm.nih.gov/>. GenBank accession number of new sequenced taxa are indicated in bold. Specie marked with * were substituted with high-related proxy (particulalry *Tamus* *communis*, *Pulmonaria* *picta*, *Helianthemum* *nummularium*, *Geranium* *rotondifolium* and *Colchicum* *lusitanicum* were substituted by *Dioscorea* *polystachya*, *Pulmonaria* *rubra*, *Helianthemum* *squamatum*, *Geranium* *pyrenaicum* and *Colchicum* *autumnale*, respectively)

| **Species** | **Family** | **GenBank accession number** | **Tot occur-rencies** | **Hay meadows Managed** | **Hay meadows Transition** | **Hay meadows Abandoned** | **Dry grassalands Managed** | **Dry grassalands Transition** | **Dry grassalands Abandoned** |
| --- | --- | --- | --- | --- | --- | --- | --- | --- | --- |
| Allium sphaerocephalon L. | Amaryllidaceae | AJ412717 | 4 | 2 | 2 | 0 | 0 | 0 | 0 |
| Allium vineale L. | Amaryllidaceae | **MH325939** | 1 | 0 | 1 | 0 | 0 | 0 | 0 |
| Angelica sylvestris L. | Apiaceae | HQ256681 | 1 | 0 | 0 | 0 | 0 | 0 | 1 |
| Bunium bulbocastanum L. | Apiaceae | HE602464 | 13 | 4 | 0 | 2 | 4 | 3 | 0 |
| Bupleurum baldense Turra | Apiaceae | AF469682 | 4 | 0 | 0 | 0 | 4 | 0 | 0 |
| Chaerophyllum aureum L. | Apiaceae | AM284409 | 4 | 2 | 1 | 1 | 0 | 0 | 0 |
| Daucus carota L. | Apiaceae | KJ415356 | 7 | 2 | 1 | 0 | 1 | 3 | 0 |
| Oenanthe pimpinelloides L. | Apiaceae | AY691935 | 3 | 2 | 0 | 0 | 0 | 1 | 0 |
| Hedera helix L. | Araliaceae | AB817808 | 2 | 0 | 0 | 2 | 0 | 0 | 0 |
| Achillea collina Becker ex Rchb. | Asteraceae | AY603198 | 19 | 1 | 8 | 0 | 8 | 2 | 0 |
| Anthemis arvensis L. | Asteraceae | EU179214 | 1 | 1 | 0 | 0 | 0 | 0 | 0 |
| Carlina acanthifolia All. | Asteraceae | AY82624 | 3 | 0 | 0 | 0 | 1 | 2 | 0 |
| Centaurea jacea L. subsp. gaudini (Boiss. & Reut.) Gremli | Asteraceae | **MH325941** | 21 | 2 | 2 | 2 | 9 | 6 | 0 |
| Centaurea nigrescens Willd. | Asteraceae | KC603918 | 23 | 4 | 10 | 1 | 3 | 4 | 1 |
| Cichorium intybus L. | Asteraceae | AY218946 | 1 | 1 | 0 | 0 | 0 | 0 | 0 |
| Cirsium tenoreanum Petr. | Asteraceae | KC969556 | 9 | 1 | 6 | 1 | 0 | 1 | 0 |
| Crepis vesicaria L. | Asteraceae | DQ221209 | 6 | 6 | 0 | 0 | 0 | 0 | 0 |
| Hieracium pilosella L. | Asteraceae | AY879161 | 1 | 0 | 0 | 0 | 1 | 0 | 0 |
| Hieracium piloselloides Vill. | Asteraceae | **MH325945** | 1 | 0 | 0 | 0 | 1 | 0 | 0 |
| Leontodon hispidus L. | Asteraceae | JF801910 | 2 | 0 | 1 | 0 | 0 | 1 | 0 |
| Leucanthemum vulgare Lam. | Asteraceae | EF577315 | 22 | 5 | 4 | 0 | 6 | 6 | 1 |
| Picris hieracioides L. | Asteraceae | KF154362 | 1 | 0 | 0 | 0 | 0 | 1 | 0 |
| Pulicaria dysenterica (L.) Bernh. | Asteraceae | FM995395 | 1 | 0 | 0 | 0 | 0 | 0 | 1 |
| Taraxacum officinal (group) | Asteraceae | JQ230979 | 2 | 2 | 0 | 0 | 0 | 0 | 0 |
| Tragopon porrifolius L. | Asteraceae | EF374210 | 3 | 3 | 0 | 0 | 0 | 0 | 0 |
| Tussilago farfara L. | Asteraceae | KJ418339 | 1 | 0 | 0 | 0 | 1 | 0 | 0 |
| Myosotis arvensis (L.) Hill | Boraginaceae | EU594649 | 6 | 6 | 0 | 0 | 0 | 0 | 0 |
| Pulmonaria picta Rouy | Boraginaceae | JX19676* | 1 | 0 | 0 | 1 | 0 | 0 | 0 |
| Arabis hirsuta (L.) Scop. | Brassicaceae | KC412269 | 2 | 0 | 1 | 0 | 1 | 0 | 0 |
| Erophila verna (L.) DC. | Brassicaceae | AY047686 | 2 | 1 | 0 | 0 | 1 | 0 | 0 |
| Thlaspi alliaceum L. | Brassicaceae | AF336156 | 1 | 1 | 0 | 0 | 0 | 0 | 0 |
| Campanula rapunculus L. | Campanulaceae | FM212738 | 9 | 4 | 0 | 0 | 5 | 0 | 0 |
| Campanula trachelium L. | Campanulaceae | DQ304572 | 1 | 0 | 0 | 0 | 0 | 0 | 1 |
| Knautia purpurea (Vill.) Borbás | Caprifoliaceae | KF993582 | 2 | 0 | 0 | 0 | 2 | 0 | 0 |
| Lonicera xylosteum L. | Caprifoliaceae | EU240714 | 1 | 0 | 0 | 0 | 0 | 0 | 1 |
| Scabiosa columbaria L. | Caprifoliaceae | AY236188 | 6 | 0 | 0 | 0 | 1 | 5 | 0 |
| Valeriana officinalis L. | Caprifoliaceae | EU796889 | 6 | 1 | 0 | 5 | 0 | 0 | 0 |
| Arenaria serpyllifolia L. | Caryophyllaceae | JN589113 | 2 | 0 | 0 | 0 | 2 | 0 | 0 |
| Cerastium brachypetalum Desp. Ex Pers. | Caryophyllaceae | **MH412925** | 3 | 0 | 0 | 0 | 3 | 0 | 0 |
| Petrorhagia prolifera (L.) P.W. Ball & Heywood | Caryophyllaceae | GU440883 | 2 | 0 | 0 | 0 | 2 | 0 | 0 |
| Stellaria media (L.) Vill. | Caryophyllaceae | KF737498 | 3 | 0 | 0 | 3 | 0 | 0 | 0 |
| Helianthemum nummularium (L.) Mill. | Cistaceae | DQ092927* | 3 | 0 | 0 | 0 | 3 | 0 | 0 |
| Colchicum lusitanicum Brot. | Colchicaceae | KC899568* | 4 | 0 | 0 | 3 | 1 | 0 | 0 |
| Convolvolus arvensis L. | Convolvulaceae | AY560274 | 13 | 6 | 4 | 1 | 0 | 1 | 1 |
| Cuscuta epithymum (L) L. | Convolvulaceae | DQ924609 | 4 | 1 | 0 | 0 | 2 | 1 | 0 |
| Sedum sexangulare L. | Crassulaceae | HE999679 | 4 | 0 | 0 | 0 | 3 | 1 | 0 |
| Juniperus communis L. | Cupressaceae | AY283435 | 8 | 0 | 0 | 0 | 0 | 1 | 7 |
| Carex flacca Schreb. | Cyperaceae | DQ998915 | 8 | 1 | 0 | 0 | 3 | 3 | 1 |
| Carex hirta L. | Cyperaceae | AY278296 | 4 | 0 | 2 | 1 | 0 | 1 | 0 |
| Tamus communis L. | Dioscoreaceae | EU808018* | 2 | 0 | 0 | 0 | 0 | 0 | 2 |
| Anthyllis vulneraria L. | Fabaceae | HM468330 | 1 | 0 | 0 | 0 | 1 | 0 | 0 |
| Astragalus monspessulanus L. | Fabaceae | **MH325940** | 1 | 0 | 0 | 0 | 1 | 0 | 0 |
| Dorycnium herbaceum Vill. | Fabaceae | AF218501 | 4 | 0 | 0 | 0 | 0 | 4 | 0 |
| Genista tinctoria L. | Fabaceae | AF351095 | 11 | 0 | 1 | 1 | 4 | 4 | 1 |
| Hippocrepis comosa L. | Fabaceae | DQ642002 | 5 | 0 | 0 | 0 | 3 | 2 | 0 |
| Lathyrus pratensis L. | Fabaceae | AY839384 | 18 | 7 | 4 | 0 | 0 | 6 | 1 |
| Lathyrus sylvestris L. | Fabaceae | AY839398 | 1 | 0 | 0 | 1 | 0 | 0 | 0 |
| Lotus corniculatus L. | Fabaceae | JN861076 | 18 | 0 | 4 | 0 | 7 | 5 | 2 |
| Medicago lupulina L. | Fabaceae | JQ858257 | 10 | 5 | 2 | 0 | 3 | 0 | 0 |
| Medicago minima (L) L. | Fabaceae | KF938693 | 1 | 0 | 0 | 0 | 1 | 0 | 0 |
| Ononis spinosa L. | Fabaceae | GQ488521 | 11 | 0 | 3 | 0 | 3 | 5 | 0 |
| Trifolium campestre Schreb. | Fabaceae | DQ312025 | 16 | 5 | 2 | 0 | 8 | 1 | 0 |
| Trifolium incarnatum L. | Fabaceae | AF004302 | 4 | 3 | 1 | 0 | 0 | 0 | 0 |
| Trifolium ochroleucum Huds. | Fabaceae | DQ312107 | 9 | 0 | 0 | 0 | 8 | 1 | 0 |
| Trifolium pratense L. | Fabaceae | AF053171 | 22 | 9 | 6 | 0 | 5 | 2 | 0 |
| Trifolium repens L. | Fabaceae | KC572140 | 2 | 2 | 0 | 0 | 0 | 0 | 0 |
| Trifolium scabrum L. | Fabaceae | DQ312215 | 7 | 0 | 0 | 0 | 7 | 0 | 0 |
| Trifolium striatum L. | Fabaceae | DQ312168 | 2 | 2 | 0 | 0 | 0 | 0 | 0 |
| Vicia bythinica (L.) L. | Fabaceae | JX506191 | 4 | 0 | 1 | 0 | 0 | 3 | 0 |
| Vicia sativa L. | Fabaceae | DQ312198 | 13 | 6 | 4 | 0 | 2 | 1 | 0 |
| Vicia sepium L. | Fabaceae | KJ486549 | 1 | 0 | 0 | 1 | 0 | 0 | 0 |
| Quercus cerris L. | Fagaceae | EU628555 | 7 | 0 | 1 | 2 | 0 | 1 | 3 |
| Geranium columbinum L. | Geraniaceae | DQ525065 | 12 | 2 | 3 | 0 | 3 | 3 | 1 |
| Geranium dissectum L. | Geraniaceae | AY944413 | 20 | 9 | 7 | 2 | 1 | 1 | 0 |
| Geranium rotundifolium L. | Geraniaceae | DQ525067* | 1 | 0 | 0 | 1 | 0 | 0 | 0 |
| Hypericum montanum L. | Hypericaceae | FJ694211 | 1 | 0 | 0 | 1 | 0 | 0 | 0 |
| Hypericum perforatum L. | Hypericaceae | GU596502 | 7 | 2 | 1 | 0 | 1 | 2 | 1 |
| Luzula campestris (L.) DC. | Juncaceae | FJ213882 | 2 | 0 | 0 | 0 | 2 | 0 | 0 |
| Clinopodium alpinum (L.) Moench | Lamiaceae | AY227141 | 4 | 0 | 0 | 0 | 4 | 0 | 0 |
| Clinopodium vulgare L. | Lamiaceae | DQ667324 | 8 | 0 | 1 | 0 | 1 | 4 | 2 |
| Prunella laciniata (L.) L. | Lamiaceae | **MH325948** | 11 | 0 | 3 | 0 | 6 | 2 | 0 |
| Prunella vulgaris L. | Lamiaceae | JQ669130 | 5 | 0 | 0 | 3 | 1 | 1 | 0 |
| Stachys germanica L. | Lamiaceae | KF529593 | 1 | 0 | 1 | 0 | 0 | 0 | 0 |
| Stachys sylvatica L. | Lamiaceae | KF529643 | 1 | 0 | 0 | 1 | 0 | 0 | 0 |
| Teucrium chamaedrys L. | Lamiaceae | JN575388 | 5 | 0 | 1 | 0 | 2 | 0 | 2 |
| Thymus longicaulis C. Presl | Lamiaceae | **MH325950** | 12 | 0 | 0 | 0 | 8 | 4 | 0 |
| Linum bienne Mill. | Linaceae | FJ169527 | 7 | 0 | 2 | 0 | 3 | 2 | 0 |
| Linum catharticum L. | Linaceae | FJ169533 | 1 | 0 | 0 | 0 | 1 | 0 | 0 |
| Linum trigynum L. | Linaceae | FJ169536 | 1 | 0 | 0 | 0 | 0 | 1 | 0 |
| Malva moschata L. | Malvaceae | EF419496 | 2 | 2 | 0 | 0 | 0 | 0 | 0 |
| Fraxinus excelsior L. | Oleaceae | EU314848 | 3 | 0 | 0 | 3 | 0 | 0 | 0 |
| Anacamptis pyramidalis (L.) Rich. | Orchidaceae | AY364870 | 3 | 0 | 1 | 0 | 0 | 2 | 0 |
| Rhinanthus minorL. | Orobanchaceae | KC480393 | 13 | 8 | 1 | 0 | 4 | 0 | 0 |
| Plantago lanceolata L. | Plantaginaceae | AF313036 | 16 | 6 | 1 | 0 | 9 | 0 | 0 |
| Plantago media L. | Plantaginaceae | AY101865 | 6 | 0 | 3 | 0 | 0 | 3 | 0 |
| Veronica arvensis L. | Plantaginaceae | KF724919 | 3 | 1 | 0 | 0 | 2 | 0 | 0 |
| Veronica chamaedris L. | Plantaginaceae | AF313003 | 7 | 1 | 1 | 2 | 1 | 2 | 0 |
| Veronica urticifolia Jacq. | Plantaginaceae | AF313011 | 2 | 0 | 1 | 0 | 0 | 1 | 0 |
| Agrostis castellana Boiss. & Reut. | Poaceae | DQ146768 | 4 | 1 | 3 | 0 | 0 | 0 | 0 |
| Anthoxanthum odoratum L. | Poaceae | KC512878 | 11 | 4 | 4 | 0 | 2 | 1 | 0 |
| Arrhenatherum elatius (L.) P. Beauv. ex J. & C. Presl | Poaceae | JF904803 | 11 | 5 | 2 | 0 | 0 | 3 | 1 |
| Brachypodium rupestre (Host) Roem. & Schult. | Poaceae | JN187623 | 26 | 0 | 4 | 2 | 1 | 10 | 9 |
| Briza media L. | Poaceae | DQ539583 | 4 | 0 | 0 | 0 | 3 | 0 | 1 |
| Bromus erectus Huds. | Poaceae | AY367907 | 19 | 0 | 2 | 0 | 10 | 6 | 1 |
| Bromus hordeaceus L. | Poaceae | AF494347 | 10 | 5 | 5 | 0 | 0 | 0 | 0 |
| Cynosurus cristatus L. | Poaceae | JQ972939 | 17 | 7 | 7 | 1 | 2 | 0 | 0 |
| Cynosurus echinatus L. | Poaceae | AF532937 | 2 | 1 | 0 | 0 | 1 | 0 | 0 |
| Dactylis glomerata L. | Poaceae | KF713216 | 37 | 9 | 10 | 5 | 4 | 6 | 3 |
| Elymus repens (L.) Gould. | Poaceae | GQ365145 | 1 | 0 | 0 | 0 | 0 | 0 | 1 |
| Festuca arundinacea Schreb. | Poaceae | AF171177 | 11 | 2 | 4 | 0 | 0 | 2 | 3 |
| Gaudinia fragilis (L.) P. Beauv. | Poaceae | DQ539600 | 1 | 1 | 0 | 0 | 0 | 0 | 0 |
| Holcus lanatus L. | Poaceae | JQ972937 | 16 | 6 | 5 | 2 | 1 | 2 | 0 |
| Lolium perenne L. | Poaceae | EF379075 | 5 | 5 | 0 | 0 | 0 | 0 | 0 |
| Phleum bertolonii DC. | Poaceae | DQ539568 | 27 | 10 | 9 | 0 | 6 | 2 | 0 |
| Poa bulbosa L. | Poaceae | EU792391 | 3 | 0 | 0 | 0 | 3 | 0 | 0 |
| Poa pratensis L. | Poaceae | JF786337 | 3 | 0 | 0 | 0 | 0 | 2 | 1 |
| Poa trivialis L. | Poaceae | GQ324555 | 17 | 5 | 7 | 3 | 1 | 1 | 0 |
| Trisetaria flavescens (L.) Baumg. | Poaceae | DQ336830 | 23 | 4 | 6 | 0 | 10 | 3 | 0 |
| Polygala flavescens DC. | Polygalaceae | **MH325946** | 1 | 0 | 0 | 0 | 1 | 0 | 0 |
| Rumex acetosa L. | Polygonaceae | FJ503011 | 5 | 4 | 0 | 1 | 0 | 0 | 0 |
| Lysimachia punctata L. | Primulaceae | AF547718 | 1 | 1 | 0 | 0 | 0 | 0 | 0 |
| Clematis vitalba L. | Ranunculaceae | GU732642 | 2 | 0 | 0 | 0 | 0 | 0 | 2 |
| Helleborus bocconei Ten. | Ranunculaceae | AJ347894 | 1 | 0 | 1 | 0 | 0 | 0 | 0 |
| Ranunculus bulbosus L. | Ranunculaceae | AM503891 | 13 | 10 | 0 | 0 | 3 | 0 | 0 |
| Ranunculus lanuginosus L. | Ranunculaceae | AY680163 | 6 | 0 | 0 | 6 | 0 | 0 | 0 |
| Ranunculus repens L. | Ranunculaceae | AY680160 | 1 | 1 | 0 | 0 | 0 | 0 | 0 |
| Agrimonia eupatoria L. | Rosaceae | AEU90798 | 5 | 0 | 1 | 2 | 0 | 1 | 1 |
| Crataegus monogyna Jacq. | Rosaceae | EU683926 | 13 | 0 | 3 | 6 | 1 | 2 | 1 |
| Filipendula vulgaris Moench | Rosaceae | AJ416467 | 1 | 1 | 0 | 0 | 0 | 0 | 0 |
| Fragaria vesca L. | Rosaceae | AF163510 | 1 | 0 | 1 | 0 | 0 | 0 | 0 |
| Potentilla hirta L. | Rosaceae | **MH325947** | 5 | 0 | 1 | 0 | 3 | 1 | 0 |
| Potentilla reptans L. | Rosaceae | PRU90784 | 18 | 8 | 4 | 3 | 0 | 2 | 1 |
| Prunus spinosa L. | Rosaceae | AF318730 | 24 | 0 | 1 | 8 | 3 | 5 | 7 |
| Pyrus communis L. | Rosaceae | JQ392467 | 2 | 0 | 1 | 1 | 0 | 0 | 0 |
| Rosa canina L. | Rosaceae | FM164423 | 20 | 1 | 3 | 6 | 0 | 4 | 6 |
| Rubus canescens DC. | Rosaceae | AY083370 | 5 | 0 | 2 | 1 | 1 | 0 | 1 |
| Sanguisorba minor Scop. | Rosaceae | AY635039 | 19 | 0 | 0 | 0 | 10 | 7 | 2 |
| Cruciata glabra (L.) Ehrend. | Rubiaceae | **MH325942** | 12 | 0 | 1 | 5 | 0 | 2 | 4 |
| Galium aparine L. | Rubiaceae | AF419192 | 5 | 0 | 1 | 3 | 0 | 0 | 1 |
| Galium mollugo L. subsp. erectum Syme | Rubiaceae | **MH325943** | 33 | 5 | 9 | 5 | 3 | 6 | 5 |
| Galium verum L. | Rubiaceae | **MH325944** | 9 | 1 | 3 | 0 | 3 | 2 | 0 |
| Sherardia arvensis L. | Rubiaceae | FJ695466 | 4 | 3 | 0 | 0 | 1 | 0 | 0 |
| Salix apennina A.K. Skwortsov | Salicaceae | **MH325949** | 3 | 0 | 0 | 3 | 0 | 0 | 0 |
| Acer campestre L. | Sapindaceae | AF401158 | 7 | 0 | 0 | 6 | 0 | 1 | 0 |
| Daphne laureola L. | Thymelaeaceae | GQ167536 | 1 | 0 | 0 | 0 | 0 | 0 | 1 |
| Viola alba Besser | Violaceae | HM851450 | 10 | 0 | 0 | 5 | 1 | 1 | 3 |
| Viola arvensis Murray | Violaceae | HM851454 | 1 | 1 | 0 | 0 | 0 | 0 | 0 |

**References**

Conti, F., Abbate, G., Alessandrini, A., Blasi, C. 2005. An Annotated Checklist of the Italian Vascular Flora. Palombi Editori, Roma

.

**Table S3.** Results of indicator species analysis of plant species through comparison between Habitat and Succession. Only species showing significant indication values (P value <0.05) are shown. See also figure 3B) for distribution of main species according to Habitat type and Stage of Succession obtained with CCA.

| **Species** | **Habitat** | | **Succession** | | **Cluster** | | **Indicator value (%)** | | | **P value** |
| --- | --- | --- | --- | --- | --- | --- | --- | --- | --- | --- |
| Ranunculus bulbosus | Hay meadows | | Managed | | I | | 77 | | | 0.001 |
| Crepis vesicaria |  |  |  |  | I | | 60 | | | 0.001 |
| Myosotis arvensis |  |  |  |  | I | | 60 | | | 0.001 |
| Lolium perenne |  |  |  |  | I | | 50 | | | 0.001 |
| Rhinanthus minor |  |  |  |  | I | | 49 | | | 0.001 |
| Geranium dissectum |  |  |  |  | I | | 41 | | | 0.003 |
| Phleum bertolonii |  |  |  |  | I | | 37 | | | 0.001 |
| Trifolium pratense |  |  |  |  | I | | 37 | | | 0.001 |
| Potentilla reptans |  |  |  |  | I | | 36 | | | 0.006 |
| Rumex acetosa |  |  |  |  | I | | 32 | | | 0.016 |
| Tragopon porrifolius |  |  |  |  | I | | 30 | | | 0.020 |
| Cynosurus cri status |  |  |  |  | I | | 29 | | | 0.019 |
| Convolvolus arvensis |  |  |  |  | I | | 28 | | | 0.032 |
| Vicia sativa |  |  |  |  | I | | 28 | | | 0.029 |
| Lathyrus pratensis |  |  |  |  | I | | 27 | | | 0.029 |
| Bromus hordeaceus |  |  |  |  | I | | 25 | | | 0.039 |
| Medicago lupulina |  |  |  |  | I | | 25 | | | 0.037 |
| Centaurea nigrescens |  |  | Transition | | II | | 43 | | | 0.001 |
| Cirsium tenoreanum |  |  |  |  | II | | 40 | | | 0.001 |
| Achillea collina |  |  |  |  | II | | 34 | | | 0.008 |
| Poa trivialis |  |  |  |  | II | | 29 | | | 0.022 |
| Dactylis glomerata |  |  |  |  | II | | 27 | | | 0.023 |
| Ranunculus lanuginosus |  |  | Abandoned | | III | | 60 | | | 0.001 |
| Acer campestre |  |  |  |  | III | | 51 | | | 0.003 |
| Valeriana officinalis |  |  |  |  | III | | 42 | | | 0.003 |
| Fraxinus excelsior |  |  |  |  | III | | 30 | | | 0.021 |
| Salix apennina |  |  |  |  | III | | 30 | | | 0.022 |
| Stellaria media |  |  |  |  | III | | 30 | | | 0.024 |
| Crataegus monogyna |  |  |  |  | III | | 28 | | | 0.026 |
| Prunus spinosa |  |  |  |  | III | | 27 | | | 0.047 |
| Viola alba Besser subp. dehnardtii |  |  |  |  | III | | 25 | | | 0.045 |
| Trifolium ochroleucum | Dry grassland | | Managed | | IV | | 71 | | | 0.001 |
| Trifolium scabrum |  |  |  |  | IV | | 70 | | | 0.001 |
| Thymus longicaulis |  |  |  |  | IV | | 53 | | | 0.001 |
| Bromus erectus |  |  |  |  | IV | | 53 | | | 0.001 |
| Sanguisorba minor |  |  |  |  | IV | | 53 | | | 0.001 |
| Plantago lanceolata |  |  |  |  | IV | | 51 | | | 0.001 |
| Trisetaria flavescens |  |  |  |  | IV | | 43 | | | 0.001 |
| Clinopodium alpinum |  |  |  |  | IV | | 40 | | | 0.004 |
| Bupleurum baldense |  |  |  |  | IV | | 40 | | | 0.003 |
| Trifolium campestre |  |  |  |  | IV | | 40 | | | 0.002 |
| Centaurea jacea subsp. gaudini |  |  |  |  | IV | | 39 | | | 0.001 |
| Prunella laciniata |  |  |  |  | IV | | 33 | | | 0.013 |
| Cerastium brachypetalum |  |  |  |  | IV | | 30 | | | 0.017 |
| Helianthemum nummularium |  |  |  |  | IV | | 30 | | | 0.015 |
| Poa bulbosa |  |  |  |  | IV | | 30 | | | 0.024 |
| Campanula rapunculus |  |  |  |  | IV | | 28 | | | 0.018 |
| Lotus corniculatus |  |  |  |  | IV | | 27 | | | 0.034 |
| Scabiosa columbaria |  |  | Transition | | V | | 42 | | | 0.005 |
| Dorycnium herbaceum |  |  |  |  | V | | 40 | | | 0.003 |
| Brachypodium rupestre |  |  |  |  | V | | 38 | | | 0.001 |
| Juniperus communis |  |  | Abandoned | | VI | | 61 | | | 0.001 |
| **Significant Indicator Distribution** |  |  |  |  |  |  |  |  |  |  |
| Cluster | I | II | | III | | IV | | V | VI | |
| Number of indicator species | 17 | 5 | | 9 | | 17 | | 3 | 1 | |

**Figure S1.** Phylogenetic tree of the plant species recorded in the 60 plots, obtained using nrDNA sequences available at the NCIB database and performing new sequences for 13 species not present in the database. The topology of phylogenetic inference was resolved at the family level in accordance with the APG IV and the evolutionary relationships of all the surveyed species were performed using a maximum likelihood (ML) community phylogeny using RAxML. The statistical support to the nodes was estimated using the bootstrap method. The phylogeny was calibrated adopting ages in Magallón et al. (2015). Numbers next to nodes indicate the bootstrap support for each node. A Newick format of the file is provided as Appendix S6.


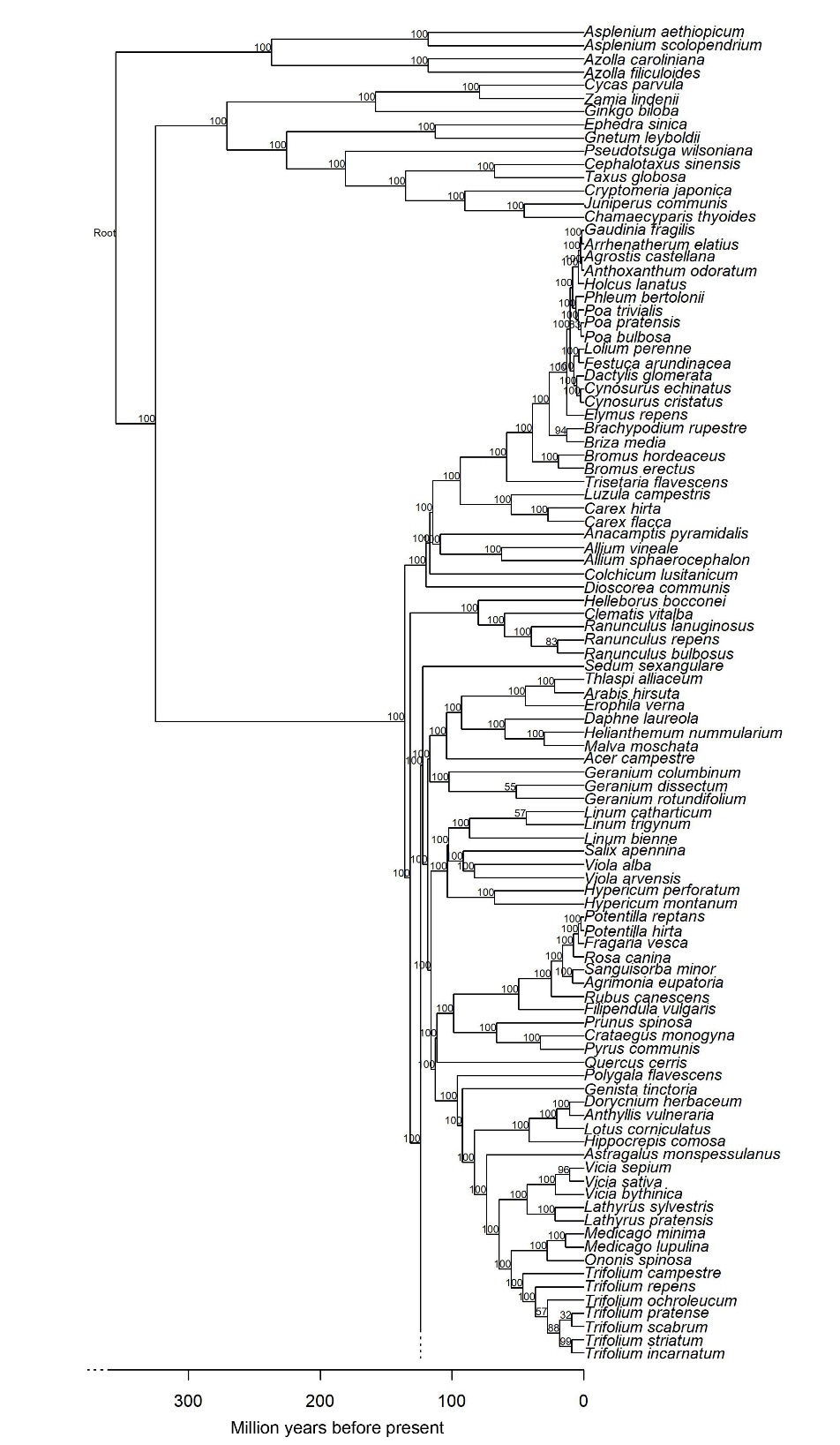


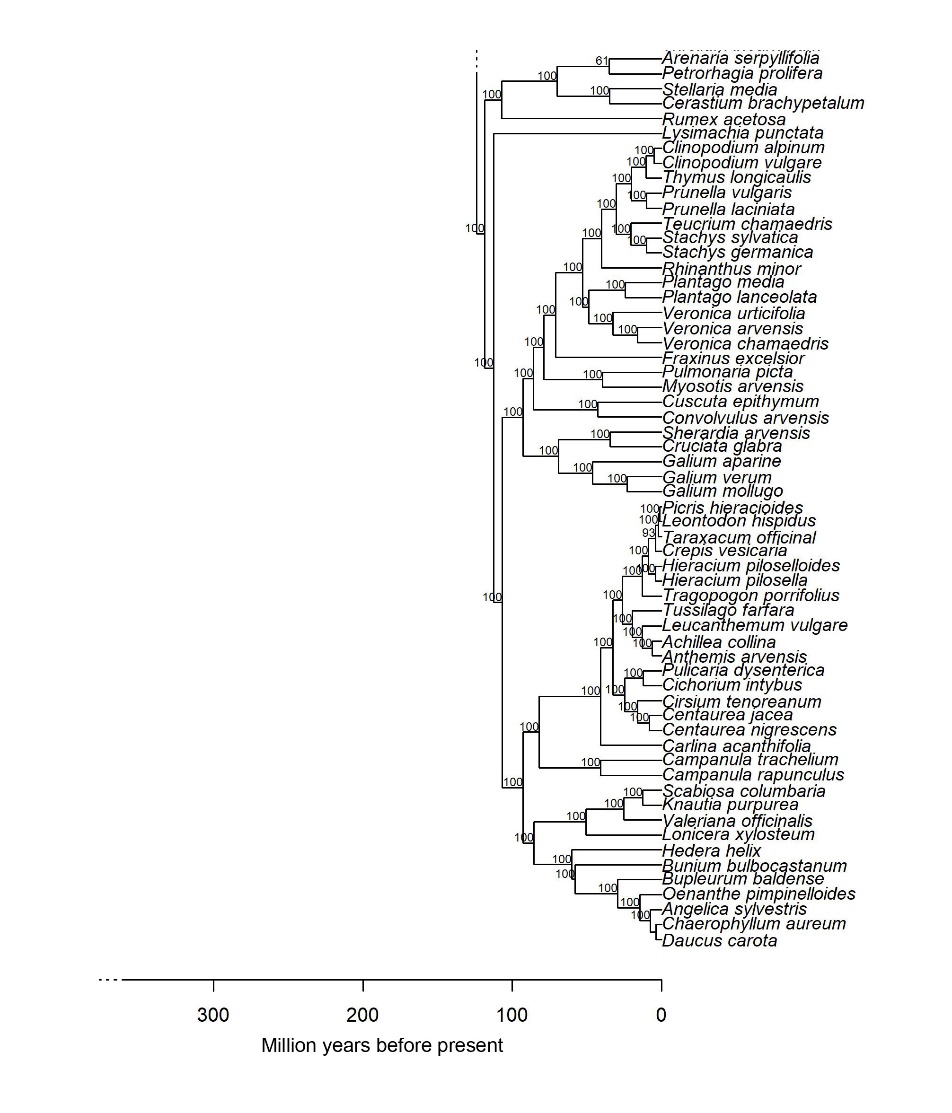


**References**

Magallón, S., Gómez‐Acevedo, S., Sánchez‐Reyes, L.L., & Hernández‐Hernández, T. 2015. A metacalibrated time‐tree documents the early rise of flowering plant phylogenetic diversity. New Phytologist, 207(2):437-453.
